# Platelet-rich plasma vs hyaluronic acid to treat knee degenerative pathology: study design and preliminary results of a randomized controlled trial

**DOI:** 10.1186/1471-2474-13-229

**Published:** 2012-11-23

**Authors:** Giuseppe Filardo, Elizaveta Kon, Alessandro Di Martino, Berardo Di Matteo, Maria Letizia Merli, Annarita Cenacchi, Pier Maria Fornasari, Maurilio Marcacci

**Affiliations:** 1 Nano-Biotechnology Laboratory, Rizzoli Orthopaedic Institute, Via di Barbiano n. 1/10, Bologna, 40136, Italy; 2 Biomechanics Laboratory, Rizzoli Orthopaedic Institute, Via di Barbiano n. 1/10, Bologna, 40136, Italy; 3 Immunohematology and Transfusion Medicine and Cell and Musculoskeletal Tissue Bank, Rizzoli Orthopaedic Institute, Via di Barbiano n. 1/10, Bologna, 40136, Italy

**Keywords:** Growth factors, PRP, HA, Intra-articular injections, Randomized, Degenerative, Knee

## Abstract

**Background:**

Platelet Rich Plasma (PRP), a blood-derived product rich in growth factors, is a promising treatment for cartilage defects but there is still a lack of clinical evidence. The aim of this study is to show, through a randomized double blind prospective trial, the efficacy of this procedure, by comparing PRP to Hyaluronic Acid (HA) injections for the treatment of knee chondropathy or osteoarthritis (OA).

**Methods:**

109 patients (55 treated with HA and 54 with PRP) were treated and evaluated at 12 months of follow-up. The patients were enrolled according to the following inclusion criteria: age> 18 years, history of chronic (at least 4 months) pain or swelling of the knee and imaging findings of degenerative changes of the joint (Kellgren-Lawrence Score up to 3). A cycle of 3 weekly injections was administered blindly. All patients were prospectively evaluated before and at 2, 6, and 12 months after the treatment by: IKDC, EQ-VAS, TEGNER, and KOOS scores. Range of motion and knee circumference changes were measured over time. Adverse events and patient satisfaction were also recorded.

**Results:**

Only minor adverse events were detected in some patients, such as mild pain and effusion after the injections, in particular in the PRP group, where a significantly higher post-injective pain reaction was observed (p=0.039). At the follow-up evaluations, both groups presented a clinical improvement but the comparison between the two groups showed a not statistically significant difference in all scores evaluated. A trend favorable for the PRP group was only found in patients with low grade articular degeneration (Kellgren-Lawrence score up to 2).

**Conclusions:**

Results suggest that PRP injections offer a significant clinical improvement up to one year of follow-up. However, conversely to what was shown by the current literature, for middle-aged patients with moderate signs of OA, PRP results were not better than those obtained with HA injections, and thus it should not be considered as first line treatment. More promising results are shown for its use in low grade degeneration, but they still have to be confirmed.

## Background

Every day, orthopaedic, sports medicine, and rheumatologic physicians face the complex issue of cartilage pathology. Its incidence [1-3] is rapidly increasing due to massive involvement in sports activity by the entire population, from the young to the middle-aged and even elderly individuals prompted by awareness of the importance of physical activity as a preventive medical approach. Despite the positive aspects of this life-style, there are also some medical problems: in particular, cartilage lesions are becoming one of the most important challenges for both basic researchers and clinicians. In fact, cartilage has limited healing potential for several reasons: first of all, the relative isolation from systemic regulation caused by the lack of nerves and vessels when compared to other tissues. Furthermore, its complex histological structure, consisting of chondrocytes surrounded by matrix made of a specialized framework of collagen, aggrecans and fluid, determines an intrinsic vulnerability that, starting from small and focal lesions, can develop into an accelerated degenerative process ending in osteoarthritis (OA), a chronic condition that is difficult to treat by conservative means. Eventually, this process often requires a sacrificing surgical approach such as metal resurfacing. Several treatments, both conservative and surgical, have been proposed to address cartilage pathology, but results are often only partially satisfactory and limited over time [4-6].

Current research is investigating new methods for stimulating repair or replacing damaged cartilage. In particular, the most recent knowledge about tissue biology concerns a complex regulation of growth factors (GFs) for the normal tissue structure and its reaction to damage. The influence of GFs on cartilage repair has been investigated *in vitro* and *in vivo*[7-10], and Platelet Rich Plasma (PRP) is a simple, low- cost and minimally-invasive method that provides a natural concentrate of autologous GFs from the blood [11]. This method is now being increasingly applied in clinical practice to treat knee degenerative pathology, such as chondropathy and early OA [12]. The biological rational of PRP is that platelets contain storage pools of GFs, cytokines, chemokines and many other mediators [13-17]. Several *in vitro* and *in vivo* animal studies have showed the potential beneficial effect of PRP in promoting cellular anabolism and tissue regeneration [7,18].

This fascinating regenerative approach has led to promising findings but also to some controversies in the scientific community [12,19-22].

Despite its widespread application, there is a lack of high level studies in the literature to demonstrate the real efficacy of PRP. We believe that it is important to have scientifically robust studies to clearly prove the real potential of this biological approach in order to guide its clinical use and avoid an indiscriminate clinical application, and therefore a high level study was designed. Due to similarity in the current treatment indications and the widespread use of viscosupplementation, this was chosen as a control group, whereas for ethical reasons a placebo group was avoided. Viscosupplementation involves the use of intra-articular injections of hyaluronic acid (HA), a glycosaminoglycan that provides joint lubrication, shock absorbency, and acts as the backbone for the proteoglycans of the extracellular matrix [4]. Although beneficial effects on pain, function and patient global assessment have been documented, the real entity of improvement and which of the many available HA products can offer the best results is not clear [23]. Even though scientific debate on these aspects is still ongoing, viscosupplementation is currently a widely used treatment for joint degeneration pathology, thus it was decided that HA would have been an appropriate control group for PRP.

This study shows the design of this prospective controlled double-blinded randomized trial and the preliminary results on the first 109 patients treated and evaluated up to 12 months of follow-up after PRP or HA injections.

## Methods

The Hospital Ethic Committee approved the study design, and patients gave their written consent to participate in this trial.

The following diagnostic criteria for patient selection were used: patients affected by a monolateral lesion with a history of chronic (for at least 4 months) pain or swelling of the knee and imaging findings of degenerative changes of the joint (Kellgren Lawrence 0 to III at X-ray evaluation or MRI findings of degenerative changes in patients presenting with no OA X-ray findings). Exclusion criteria were: age > 80 years; Kellgren-Lawrence score > 3; systemic disorders such as diabetes, rheumatoid arthritis, major axial deviation (varus >5°, valgus > 5°), haematological diseases (coagulopathy), severe cardiovascular diseases, infections, immunodepression, patients in therapy with anticoagulants or antiaggregants, use of NSAIDs in the 5 days before blood donation and patients with Hb values < 11 g/dl and platelet values < 150,000/mm3.

Patients were prospectively evaluated basally and at 2, 6, and 12 months of follow-up using IKDC, KOOS, EQ-VAS for general health status, and Tegner scores. Furthermore, at basal evaluation and at every follow-up the ROM and the transpatellar circumference of both the index knee and the contralateral one were measured to check if any changes occurred over time. Patient satisfaction and adverse events were also reported. All the clinical evaluations were performed by a medical member of staff not involved in the injective procedure, to keep the study double blinded. At the end of the study, the nature of the injected substance was revealed to the patients.

A power analysis was performed for the primary endpoint of IKDC subjective score improvement at the 12-month follow-up for PRP. From a pilot study, a standard deviation of 15.2 points was found. With an alpha error of 0.05, a beta error of 0.2 and a minimal clinically significant difference of 6.7 points corresponding to 1/3 of the documented mean improvement, the minimum sample size was 83 for each group. Considering a possible drop out of 15%, 96 patients are required for a total of 192 patients. At present, 109 patients have reached the 12 month follow-up; among these patients, 54 were treated with 3 autologous PRP intra-articular injections, and 55 were treated with 3 HA (>1500 KDa; Hyalubrix®, Fidia, Abano Terme (PD), Italy) injections. The groups were homogeneous for sex, age, BMI, symptoms duration and previous treatments (Table 1); therefore, a comparative analysis to have preliminary indications could be done.

**Table 1:** The two treatment groups are homogeneous for all the parameters evaluated

	**Comparative demographics**		
	**PRP**	**HA**	
**N. of patients**	54	55	
**Age**	55	58	N.S.
**Sex**	37 M, 17 F	31 M, 24 F	N.S.
**BMI**	27	26	N.S.
**Symptoms**	64 months	63 months	N.S.
**Kellgren**	2.2	2.1	N.S.
**Prev treat**	7 no	5 no	N.S.
	13 conservative	21 conservative	
	34 surgical	29 surgical	
**Basal IKDC**	50.2 ± 15.7	47.4 ± 14.0	N.S.

To ensure the blinding of the patients, all of them underwent blood harvesting to obtain autologous PRP which was used only in half of them, according to a randomization list (the randomization list, provided by an independent statistician, was kept in a dedicated office that was contacted by the physician by a phone call just before the injective procedure for the communication of the treatment allocation). The procedure consisted of a 150-ml venous blood sample for every knee treated. Then, 2 centrifugations (the first at 1480 rpm for 6 minutes to separate erythrocytes, and a second at 3400 rpm for 15 minutes to concentrate platelets) produced a unit (20 ml) of PRP. The unit of PRP was divided into 4 small units of 5 ml each. One unit was sent to the laboratory for analysis of platelet concentration and for a quality test, whereas 3 units were stored at −30°C. Freezing them allows the time to proceed with the quality analysis, and this could be considered as an advantage since it increases the safety of the procedure, thus ensuring a controlled not contaminated intra-articular delivery of the product. However, it has to be underlined that this could also be considered as a disadvantage: the alteration of the morphology and decrease in platelet functional properties, which includes degranulation of alpha-granules, after storing platelets in freezing conditions, is known. However, there are no data on the effect of freezing on the clinical results of platelet injections, and freeze-thawing is even one of the methods used for releasing intracellular GFs. Having freshly-harvested PRP might preserve all the platelet functions better, but currently the data are still controversial and it is uncertain whether freeze-thawing adversely affects their properties to the point of impairing their clinical efficacy.

One week after blood harvesting the injection cycle was started, with 3 weekly injections of PRP or HA. At the time of injection the syringe was appropriately covered to prevent patients from discovering the substance they were receiving. In the case of PRP administration, the total number of platelets per milliliter in the PRP represented a mean increase of 5 times compared with whole blood values. In the platelet concentrate the presence of leukocytes was also observed, with a concentration of 1.2 times with respect to the normal blood value.

After the injection, patients were sent home with instructions to restrict the use of the leg for at least 24 hours and to use cold therapy/ice on the affected area to relieve pain. During this period, the use of non-steroidal medication was forbidden. During the treatment period, rest or mild activities (such as using an exercise bike or mild exercise in a pool) were permitted, and subsequently a gradual resumption of normal sport or recreational activities was allowed, as tolerated.

### Statistical analysis

All continuous data were expressed in terms of the mean and the standard deviation of the mean. One Way ANOVA was performed to assess differences among groups when the Levene test for homogeneity of variances was not significant (p<0.05); otherwise, the Mann Whitney test (2 groups) or the Kruskal Wallis test (more than 2 groups) were used. The Least Significant Difference test was performed as a post-hoc pair-wise analysis of the Kruskal Wallis test. The generalized linear model for repeated measures with Sidak correction for multiple comparisons was performed to test differences of the scores at different follow-up times. The non-parametric Pearson’s Chi square test evaluated by Exact methods was performed to investigate the relationships between grouping variables. For all tests, p<0.05 was considered significant. Statistical Analysis was carried out by using the Statistical Package for the Social Sciences (SPSS) software version 15.0 (SPSS Inc., Chicago, USA).

## Results

No major complications related to the injections were observed during the treatment and follow-up period.

When comparing the two treatments, a significantly higher post-injective pain reaction was observed in the PRP group (p=0.039) (Table 2). However, this reaction was self-limiting within a few days and did not compromise the overall outcome.

**Table 2:** The PRP group showed a significantly higher post injective pain reaction (p=0.039)

**Level of pain and swelling: comparison**			
**Pain**		**Swelling**	
**(n. of days x level 1–10)**		**(n. of days x level 1–10)**	
**PRP**	**HA**	**PRP**	**HA**
16.7	9.2	10.3	7.2
PRP **>** HA		PRP **=** HA	

**Figure 1:**
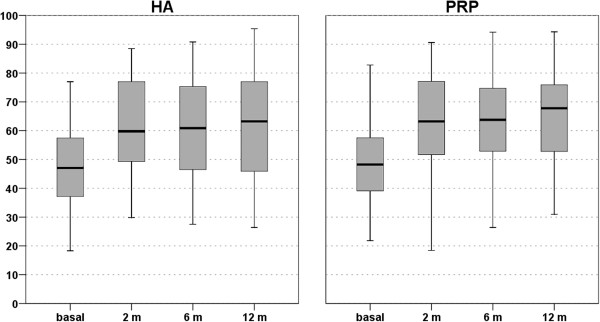
**IKDC subjective score in both PRP and HA treatment groups before and at 2, 6, and 12 months after treatment.** No statistical inter-group difference was observed.

**Figure 2:**
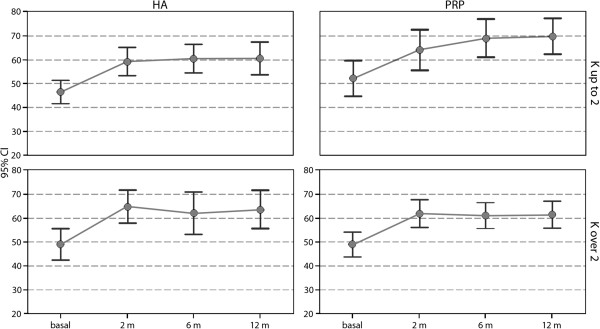
IKDC subjective results obtained with PRP and HA treatments in two patient subgroups: patients affected by Kellgren Lawrence grade 0-II lesions and patients presenting grade III level of knee degeneration.

In fact, preliminary analysis revealed a statistically significant improvement of all clinical scores from basal evaluation to the 2-, 6-, and 12-month follow-ups in both treatment groups. However, no differences could be detected between groups in the clinical scores evaluated. ROM and knee circumference measurements were also comparable at the different follow-up times. In particular, in the PRP group the IKDC subjective score increased from 50.2 ± 15.7 at the basal evaluation to 62.8 ± 17.6 at 2 months, 64.3 ±16.4 at 6 months, and 64.9 ± 16.8 at 12 months. In the HA group the IKDC score increased from 47.4 ± 15.7 at the basal evaluation to 61.4 ± 16.2 at 2 months, 61.0 ± 18.2 at 6 months, and 61.7 ± 19.0 at 12 months (Figures 1 and 2). The EQ-VAS presented the same trend with improvements in both groups but without any inter-group difference. The activity level, evaluated by the Tegner score, also showed a similar improvement for the PRP group (from basal 2.9 ±1.4 to 3.8 ± 1.3 at 12 months of follow-up) and HA group (from basal 2.6 ± 1.2 to 3.4 ± 1.6 at 12 months of follow-up). Finally, similar results were documented using the KOOS score, in all sub-categories: detailed data are shown in Table 3.

**Table 3:** KOOS score in all five subcategories before and at 2, 6, and 12 months after the treatment

**KOOS score trend in both treatment groups**					
**KOOS**		**BASAL**	**2 MONTHS**	**6 MONTHS**	**12 MONTHS**
**Symptoms**	**PRP**	64.0+−17.9	71.9+−17.0	73.0+−18.3	71.3+−17.9
	**HA**	67.8+−15.7	71.6+−16.3	74.3+−16.0	74.2+−17.5
**Pain**	**PRP**	65.4+−17.7	73.1+−21.5	74.2+−19.6	74.0+−19.4
	**HA**	63.1+−17.4	71.1+−18.6	73.2+−18.1	74.0+−19.4
**Activity of daily life**	**PRP**	69.9 +− 20.0	81.2+−17.9	79.1+−19.0	77.9+−20.6
	**HA**	67.8+−21.0	78.2+−17.4	77.3+−18.6	77.3+−19.8
**Sport**	**PRP**	37.6+−24.7	48.8+−25.9	48.7+−29.5	47.4+−28.2
	**HA**	34.2+−23.9	45.0+−24.1	44.7+−27.8	46.6+−27.9
**Quality of life**	**PRP**	34.9+−18.8	48.3+−22.6	48.0+−23.1	50.5+−22.6
	**HA**	33.6+−18.0	45.5+−23.9	48.5+−24.7	49.2+−26.0

No statistical difference was reported in any subcategory between groups.

**Figure 3:**
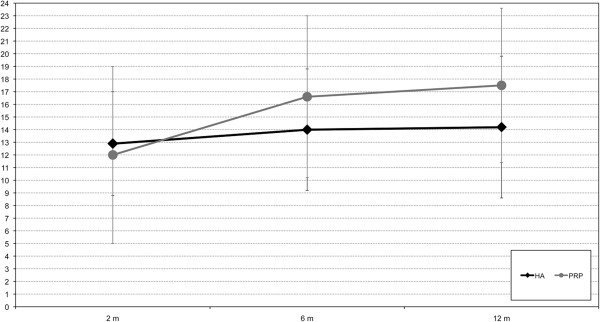
**IKDC subjective improvement from basal level to 2, 6, and 12 months of follow-up after treatment with PRP or HA injections in low grade cartilage degenerative pathology.** A tendency towards better results for PRP in this patient category is observed at both 6 and 12 months: p = 0.08 and p = 0.07, respectively.

Interestingly, despite the similar general findings, further analysis revealed slightly different results in patients affected by different degrees of cartilage degeneration. In fact, whereas PRP and HA could provide the same outcome in knees with Kellgren Lawrence III level, less degenerated joints showed a different trend, with a tendency toward better results in the PRP group at 6 and 12 months of follow-up, albeit without reaching statistical significance (p=0.08 and p=0.07, respectively) (Figure 3).

Three failures occurred, all in the HA group: in two cases with suspected intolerance to some components of HA, and so the injective treatment was stopped after the first administration. In the third case the patient was still complaining of intense pain and functional deficit and sought other treatment in another medical center.

## Discussion

Despite its wide clinical application, only a few reports have documented results for PRP in the treatment of knee degenerative lesions in the literature [24-34].

In 2008, Sanchez et al. published a retrospective observational study involving 60 patients [25], 30 treated with knee intra-articular injections of PRGF and 30 with injections of HA. Patients from both groups underwent 3 weekly injections and were evaluated basally and at 5 weeks of follow-up using the WOMAC score. Results were encouraging, even though the short follow-up is a weak point of the study. In 2010, Sampson et al. published a study [26] on 14 patients with clinical and radiographic signs of OA and previous unsuccessful conservative management (12 men and 2 women), who received 3 PRP injections 1 month apart. Evaluation was carried out up to 52 weeks using the “Brittberg-Peterson Visual Analog Pain, Activities and expectation Score”, VAS for pain, and KOOS Score: the authors found a statistically significant improvement in the scores examined, with a reduction in pain both at rest and during physical activity and, at 1 year of follow-up, 8 patients were still completely satisfied with the treatment received. In the same year, Wang-Saegusa et al. [27] published a prospective study on 261 patients with uni- or bilateral knee OA, symptomatic for more than 3 months. Patients received 3 injections 2 weeks apart, and clinical evaluation was conducted at 6 months using the WOMAC score, VAS, Lequesne Index and SF-36. Statistical analysis revealed significant results with an improvement in all the scores adopted. Despite the high number of patients evaluated, the absence of a control group is a limiting factor in the conclusions of this study. Napolitano et al. [28] treated 27 patients, either affected by simple chondropathy or initial OA, with 3 injections of 5 ml PRP performed one week apart from each other, and evaluated them up to 6 months with NRS scale for pain and WOMAC score. Significant results were obtained after treatment without occurrence of adverse events. The group led by Gobbi [29] published a preliminary report on a group of 50 active patients treated with 2 PRP injections one month apart, showing good results at up to 1 year of follow-up both in patients who had or had not undergone previous operative intervention for cartilage lesions. A study by Spakova et al. [30] compared the efficacy of PRP versus viscosupplementation on a cohort of 120 patients divided into two treatment groups and evaluated through the WOMAC score and a pain numeric rating scale. At 3 and 6 months of follow-up an increase in the clinical score was observed in both groups with statistically superior results in PRP group.

The present authors previously performed studies to evaluate this clinical application of PRP, and recorded safety and interesting findings [31-34]: the first one was a prospective study published in 2009 [31] on 91 patients (for a total of 115 knees) treated with three injections of PRP (1 every 3 weeks). Patients underwent clinical evaluation at basal level and at 2, 6, and 12 months of follow-up. Eighty percent of the patients treated expressed satisfaction with the treatment received. The clinical outcome revealed a statistically relevant improvement in all the variables considered just 2 months after the end of treatment. These results were later confirmed at 6 months of follow-up, whereas a tendency to worsen was reported after 6 to 12 months of follow-up. Some influencing factors were detected: in particular it was observed that young male patients were the best responding group, especially in case of simple chondropathy without signs of OA. A later study [32] evaluating the same patients at 24 months of follow-up confirmed this trend with a further decrease in the clinical outcome, thus concluding that intra-articular therapy with PRP is time dependent with an average duration of 9 months and better and longer lasting results are achieved in younger patients with lower levels of joint degeneration. In another multi-center study [33], the clinical effectiveness of PRP was compared to low molecular weight HA (LWHA) and high molecular weight HA (HWHA). Three homogeneous groups of patients were respectively treated with 3 injections of PRP, LWHA, or HWHA. The results highlighted a better performance for PRP group at 6 months of follow-up. In particular, subgroup analysis (chondropathy vs early vs severe OA) revealed that in the chondropathy group PRP gave markedly better results than HA at 6 months of follow-up, whereas in the early OA group the gap in favor of PRP was reduced and in the severe OA subgroup no difference in clinical outcome was observed between treatments. Furthermore, patients under 50 years old have a greater chance to benefit from this biological approach with this GFs supplementation. Finally, the present authors recently published a comparative study between PRP with or without leukocytes used to treat 144 patients affected by knee cartilage pathology, and showed comparable positive clinical effects with both treatments, with PRP-leukocyte group suffering from more swelling and pain reaction after the injections [34].

Despite the increasing number of reports, there is a lack of high level studies: only one randomized clinical trial has been recently published [35]. This is a multicenter study led by Sanchez who compared at short-term follow-up PRGF (a single spinning procedure giving a leukocyte free PRP with a low platelet concentration) with HA administered on a weekly basis for the treatment of symptomatic knee OA. With these treatments, adverse events were mild and evenly distributed between the PRP and HA groups. The rate of response to PRGF was higher in all outcome measures, although no significant differences were found; however, compared with the rate of response to HA, the rate of response to PRGF (primary outcome measure) was 14.1 percentage points significantly higher.

In the present authors’ studies a different platelet concentrate was used, obtained through a double-spinning procedure which provides many platelets but also leukocytes. Cellularity is one of the most debated aspects when evaluating PRP properties and the results obtained with its application. In fact, not only platelets but also leukocytes, monocytes, macrophages, and mast cells are contained in many platelet concentrates. Some authors define PRP as only platelets and attribute better results to leucocyte depletion, because of the deleterious effects of proteases and reactive oxygen released from white cells; others consider them as a source of important cytokines and enzymes, that may be important also for the prevention of infections, and report that PRP significantly inhibits the growth of Staphylococcus Aureus and Escherichia Coli [7].

This is the first double-blind randomized clinical trial available in the literature on this kind of double-spinning high concentrate leukocyte PRP, and more in general it is also the first randomized trial with such a high number of patients evaluated at 12 months of follow-up. For these reasons, despite being preliminary with respect to the objective of the entire study, the present results on the evaluated cohorts of patients are already significant representing up to now one of the highest available evidence on PRP use for knee degenerative pathology, and already give us some indications on the potential of this biological approach.

The analysis of the results obtained with this randomized trial has already underlined important aspects. The safety and the significant clinical improvement of this procedure were confirmed. A higher pain reaction after PRP injection was found, probably due to the leukocyte content of our platelet concentrate, but without jeopardizing clinical results up to 1 year follow-up. Conversely to what was shown by the current literature of comparative studies [25,30,33,35], PRP did not offer better results compared to HA in this series. However, it has to be emphasized that the average age of the enrolled patients was higher than those of other studies and the present authors previously observed a worse outcome for older patients treated with PRP [31,32], and a sub-analysis of different patient categories showed some interesting findings. In fact, a tendency towards better results at both 6 and 12 months of follow-up in favor of PRP treatment was seen for the less degenerated cases. It is important to consider that this is a subanalysis, and therefore the sample size is smaller and less supportive of the preliminary results found. The completion of the entire planned 192 patient evaluation will confirm whether this trend will reach a statistical and clinical significance, thus demonstrating a clear indication for this biological treatment approach, as well as the potential of the double spinning high concentrate leukocyte PRP with respect to the single spinning low concentrate leukocyte free PRP that recently showed better results with respect to HA [35].

For now, the results of this trial suggest a possible effect of this platelet concentrate on the treatment of knee degenerative pathology, with a clinical subjective improvement but not significantly better results with respect to HA; a tendency towards better improvement only in patients affected by earlier degrees of knee degeneration was observed, thus suggesting that the clinical application of PRP should be mainly restricted to this patient subgroup, whereas the indication of this treatment for high grade degeneration is lower. Due to the not significantly better results with respect to HA, PRP cannot be considered as the first line of treatment for knee OA and should be therefore restricted to patients who do not benefit from other conservative or injective treatments such as HA or, if used as first line treatment, it should be mainly targeted to patients affected only by early degrees of knee cartilage degeneration.

## Conclusion

PRP is a new treatment for knee degenerative pathologies and an increasing number of studies show promising results. However, despite its wide application in the clinical practice and the positive findings reported, there is a lack of scientific background to guide the clinical application of PRP. To avoid indiscriminate and inappropriate use of PRP, it is important to determine the type of patients who will not benefit from this treatment, and therefore the authors designed a high level study to understand clearly the indication of this treatment. The present results suggest, conversely to what was shown by the current literature, that for middle-aged patients with moderate signs of OA, PRP did not offer better results compared to HA, and thus it should not be considered as first line treatment. More promising results are shown for its use in less degenerated cases, but they still need to be confirmed.

## Competing interests

All the authors declare that they have no competing interests.

## Authors’ contributions

EK and ADM were responsible for patients' enrollment and injective treatment; BDM and MLM were involved in patients' evaluation at the established follow-ups; GF was responsible for data analysis; GF and BDM were responsible for writing the paper; AC and PMF took care of PRP production and storage; MM performed the final revision of the article. All the authors read and approved the final manuscript.

## Pre-publication history

The pre-publication history for this paper can be accessed here:

http://www.biomedcentral.com/1471-2474/13/229/prepub
